# Paroxysmal hypotension and hypoxaemia caused by severe dynamic secondary mitral and tricuspid regurgitation after acute myocardial infarction

**DOI:** 10.1093/eschf/xvaf014

**Published:** 2026-01-08

**Authors:** Weiya Li, Si Wang, Yujia Liang, Xin Wei, Mao Chen

**Affiliations:** Department of Cardiology, West China Hospital, Sichuan University, No. 37 Guoxue Road, Chengdu 610041, China; Department of Cardiology, West China Hospital, Sichuan University, No. 37 Guoxue Road, Chengdu 610041, China; Department of Cardiology, West China Hospital, Sichuan University, No. 37 Guoxue Road, Chengdu 610041, China; Department of Cardiology, West China Hospital, Sichuan University, No. 37 Guoxue Road, Chengdu 610041, China; Department of Cardiology, West China Hospital, Sichuan University, No. 37 Guoxue Road, Chengdu 610041, China

**Keywords:** Paroxysmal hypotension and hypoxaemia, Acute myocardial infarction, Mitral regurgitation, Transcatheter edge-to- edge repair

## Abstract

A 57-year-old male patient developed paroxysmal hypotension and hypoxaemia during endotracheal extubation post-acute myocardial infarction revascularization. The underlying cause was severe dynamic mitral regurgitation (MR) and secondary tricuspid regurgitation. After successful extubation and weaning, the patient’s MR nearly resolved. Discharged in improved condition with guideline-directed medical therapy, the patient was soon readmitted for heart failure with pulmonary oedema. The transthoracic echocardiogram confirmed recurrent, worsening MR. Transcatheter edge-to-edge repair was successfully performed, reducing the patient’s severe MR to trivial and resolving tricuspid regurgitation completely. At 1-year follow-up, the patient remained stable with improved cardiac function and no readmissions for cardiac causes.

## Introduction

Severe mitral regurgitation (MR) following acute myocardial infarction (AMI) encompasses two subtypes: papillary muscle partial or complete rupture and secondary MR (SMR).^1,2^ Although the incidence of MR after AMI is low, it is frequently accompanied by haemodynamic instability, recurrent pulmonary oedema, and challenges in medical management, with persistently high mortality rates in decades.^2,3^ Mitral regurgitation can lead to cardiogenic shock during the acute phase, and SMR accounts for nearly 50% of these cases.^4^ Typically, in patients with SMR without papillary muscle rupture, revascularization with primary percutaneous coronary intervention can significantly improve the severity of MR and should be considered as first-line therapy; most patients experience alleviation of regurgitation through reperfusion.^2,4^ Furthermore, current research on SMR has focused primarily on patients with chronic myocardial ischaemia after AMI or chronic heart failure.^5–7^ This article reports a case of a patient who achieved successful revascularization after AMI. Initially, the patient had no concurrent valvular regurgitation; however, subsequent development of recurrent severe SMR led to paroxysmal hypotension and hypoxaemia, which resulted in extubation failure during the patient's hospitalization in the intensive care unit. Additionally, we will elaborate in detail on the long-term prognosis of this patient.

## Case report

**Figure 1:**
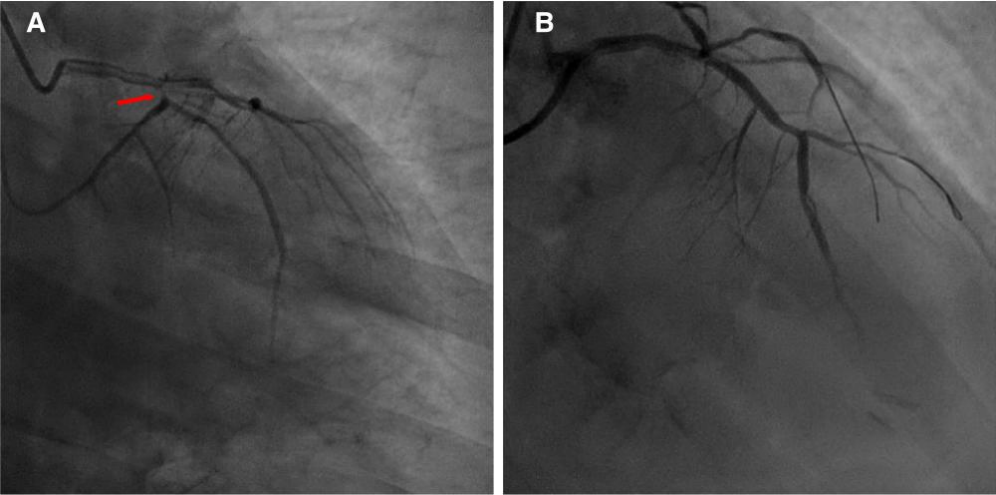
Coronary angiography showed a 95% stenosis in the circumflex branch of the coronary artery (A, indicated by the red arrow), as well as the image after circumflex revascularization supported by an intra-aortic balloon pump (B)

A 57-year-old man, experiencing 9 h of chest pain and cardiogenic shock, was intubated outside the hospital. Emergency coronary angiography revealed a 95% stenosis in the circumflex artery opening (*Figure 1A*, red arrow) and slow coronary flow in the anterior descending artery. Subsequently, he underwent coronary angioplasty (*Figure 1B*) and intra-aortic balloon pump implantation. Echocardiography indicated hypokinesia in the left ventricular posterior wall, without valve regurgitation.

**Figure 2:**
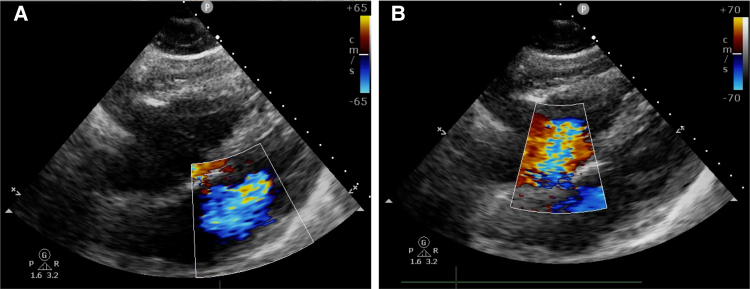
When attempting to extubate, the patient developed hypotension and hypoxaemia, with transthoracic echocardiography showing severe mitral and tricuspid regurgitation (A and B)

**Figure 3:**
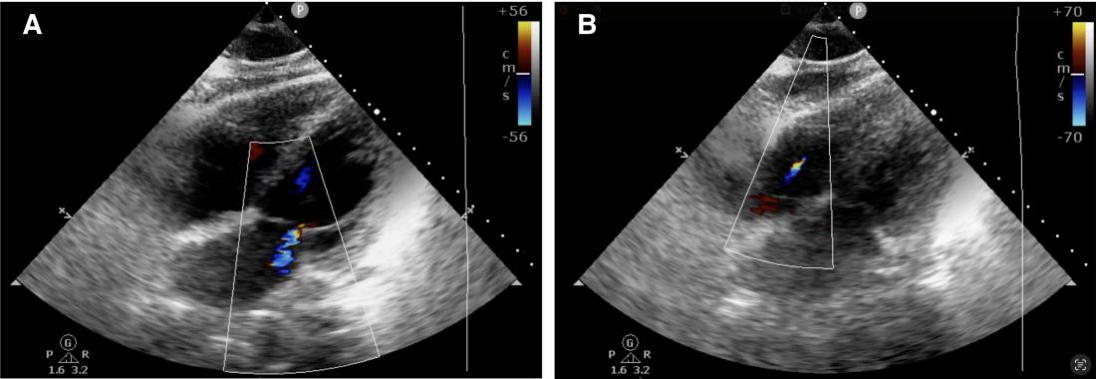
Transoesophageal echocardiography showed severe mitral regurgitation and tricuspid regurgitation nearly resolved after re-administration of sedation and analgesia (A and B)

**Figure 4:**
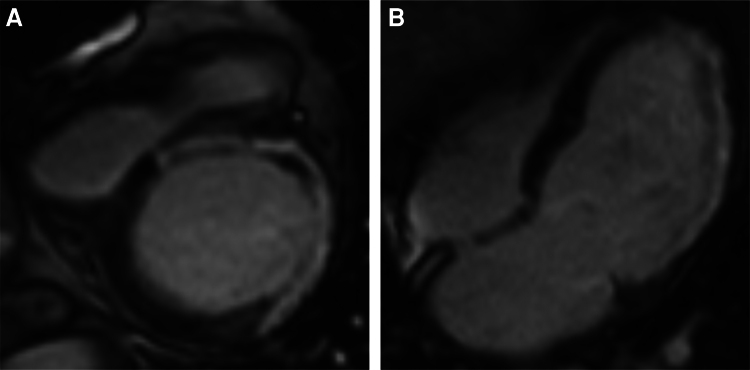
Cardiac magnetic resonance imaging showed delayed enhancement in the left ventricular septum, anterior wall, lateral wall, and apex (A and B)

Upon attempted extubation following cessation of sedatives and analgesics, the patient became agitated, aspirated pink foam sputum, and exhibited decreased oxygen saturation (<90%) and arterial blood pressure (60/40 mm Hg). Severe MR and tricuspid regurgitation (TR) were observed (*Figure 2A* and *B*, Videos 1 and 2). Intriguingly, after re-administration of sedation and analgesia, blood pressure and oxygen saturation normalized, and MR as well as TR nearly resolved (*Figure 3A* and *B*, Videos 3 and 4). Transoesophageal echocardiography revealed no valve structural abnormalities. Despite a second failed extubation due to the same issue, successful extubation was ultimately achieved under deep anaesthesia. Cardiac magnetic resonance imaging showed delayed enhancement in the left ventricular septum, anterior wall, lateral wall, and apex (*Figure 4A* and *B*).

**Figure 5:**
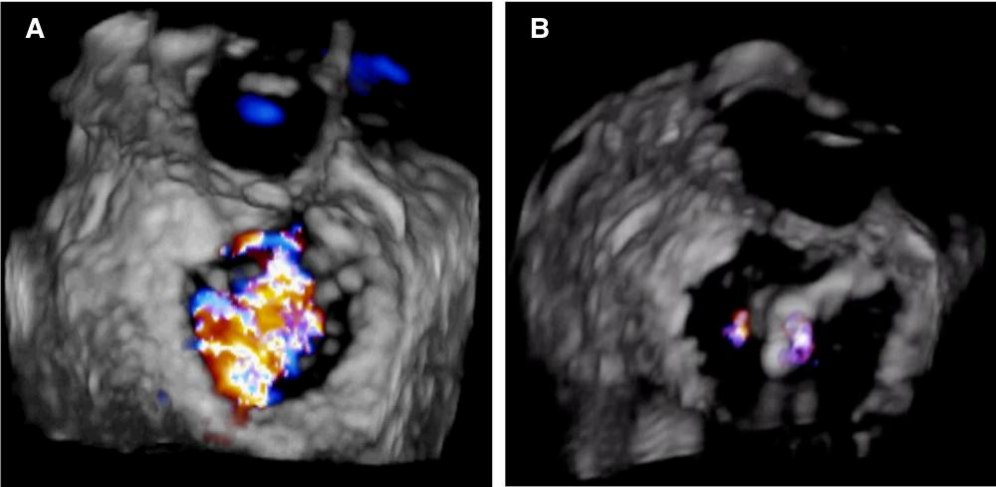
Severe mitral regurgitation before transcatheter edge-to-edge repair (A), only mild mitral regurgitation reserved after transcatheter edge-to-edge repair with MitraClip (B)

Despite ongoing guideline-directed medical therapy, the patient was readmitted 5 days post-discharge due to reduced exercise tolerance, orthopnoea, and bilateral lower extremity oedema following a cold. A transthoracic echocardiogram showed recurrent severe MR, and chest computed tomography indicated pulmonary oedema. After heart failure treatment, our multidisciplinary cardiac team decided to perform transcatheter edge-to-edge repair (TEER) with MitraClip at 1 month post-AMI. Postoperatively, MR improved from severe to extremely mild, with only trivial TR remaining (*Figure 5A* for pre-operation and *Figure 5B* for post-operation, Videos 5 and 6). Over the subsequent year, multiple follow-up echocardiographic assessments all demonstrated mild MR on this patient. The New York Heart Association functional classification and quality of life notably improved, with no further cardiac readmission events.

## Discussion and conclusion

Clinically, dynamic SMR is characterized by recurrent pulmonary oedema and difficulties in medical management due to poor drug tolerance, which can lead to cardiogenic shock.^2^ Early recognition and intervention for severe SMR are crucial for conserving healthcare resources and reducing complications and organ failure. However, current approaches for identifying dynamic SMR remain underutilized, and debates persist regarding the optimal stress assessment modality and clear timing for intervention.^8,9^ Recently, based on a three-dimensional echocardiographic study, Kimura *et al*. found that the most plausible pathogenesis of SMR is not papillary muscle displacement but rather papillary muscle separation and excessive increase in papillary muscle angle. These changes lead to mitral valve morphological deformation and are associated with acute regional ventricular wall motion abnormalities, with no presence of global left ventricular remodelling.^5^ Existing studies on SMR have focused on the long-term effects of chronic myocardial ischaemia. In this case report, we document a classic instance of acute dynamic SMR following successful revascularization for AMI, which was identified via echocardiography in an intensive care unit setting. During the endotracheal extubation of this patient, recurrent hypotension and hypoxaemia occurred. This might be attributed to the patient's agitation upon regaining consciousness, which increased resistance. It is also likely that the significantly elevated left ventricular end-diastolic pressure exacerbated the mitral valve dyssynchrony, thereby redirecting blood flow and leading to severe MR. This clinical diagnostic and therapeutic process suggests that dynamic SMR should be considered a potential aetiological factor when investigating the causes of extubation failure in critically ill patients with cardiogenic shock.

After the patient's first discharge, an episode of upper respiratory tract infection caused increased systemic metabolism and oxygen demand, which in turn precipitated the recurrence of heart failure and subsequent readmission. This clinical course prompted us to initiate TEER treatment for the patient. When the body's oxygen demand increases, the human body regulates circulatory function by activating the sympathetic nervous system and suppressing vagal tone.^10^ The subsequently released catecholamines act on the β-receptors of the heart, enhancing myocardial contractility and accelerating heart rate. These effects directly or indirectly lead to excessively high left ventricular systolic pressure. Meanwhile, catecholamines also stimulate the α-receptors of blood vessels, causing vasoconstriction and an increase in left ventricular afterload.^11^ All these factors may subject the mitral valve to greater pressure during closure and exacerbate the incoordination of mitral valve movement, thereby worsening SMR. Catecholamines can also induce pulmonary vasoconstriction, which may synergize with MR to elevate pulmonary circulatory pressure and subsequently trigger TR. In this case, secondary mitral valve motion disorder was likely the primary cause of the patient’s clinical manifestations, while TR was a secondary finding. Consequently, following the correction of MR via TEER, TR was resolved concurrently. Although successful reduction of MR with TEER has been shown to affect mortality and heart failure hospitalization rates in some studies, the controversy surrounding TEER in dynamic SMR persists.^12^ This case emphasizes that persistent hypotension and hypoxaemia following adequate revascularization and cardiac support after AMI may indicate severe dynamic SMR; MitraClip intervention could be considered. However, the optimal timing for TEER requires further discussion. Specifically, in this case, whether TEER should be performed immediately when the patient is stabilized in the intensive care unit to prevent life-threatening events from recurring due to severe dynamic SMR later on.

The recent focus seminar from the American College of Cardiology similarly emphasize that patients with severe MR following AMI who require a cardiac assist device to maintain clinical stability can be treated with TEER at an experienced centre or with surgical intervention.^2^ Considering that only a small percentage of patients with severe MR are deemed eligible for mitral valve surgery in most high-risk surgical scenarios, it may be more appropriate to proactively consider the immediate referral of these cases to an experienced TEER centre. However, the optimal timing for TEER in patients with dynamic SMR still requires further research to be clearly established—this underscores the importance of delivering timely and optimal care to these patients.

## Author contributions

W.L.: writing—original draft, data curation, conceptualization. S.W.: writing—original draft, data curation, conceptualization. Y.L.: writing—review & editing. X.W.: writing—review & editing, conceptualization. M.C.: writing—review & editing, data curation, conceptualization. All authors had access to the data and a role in writing the manuscript.

## Declarations

### Data Availability

The datasets used during the current study are available from the corresponding author on reasonable request.
